# Endocan as a marker of microvascular inflammation in kidney transplant recipients

**DOI:** 10.1038/s41598-018-37975-9

**Published:** 2019-02-12

**Authors:** Yu Ho Lee, Se-Yun Kim, Haena Moon, Jung-Woo Seo, Dong-Jin Kim, Seon Hwa Park, Yang-Gyun Kim, Ju-Young Moon, Jin Sug Kim, Kyung-Hwan Jeong, Sung-Jig Lim, Chan-Duck Kim, Jae Berm Park, Byung Ha Chung, Yeong Hoon Kim, Jaeseok Yang, Hyung-In Yang, Kyoung Soo Kim, Sang-Ho Lee

**Affiliations:** 1 Division of Nephrology, Department of Internal Medicine, Kyung Hee University, Seoul, South Korea; 2 Department of Pathology, Kyung Hee University, Seoul, South Korea; 3 Division of Nephrology, Department of Internal Medicine, Kyungpook National University Hospital, Daegu, South Korea; 4 Department of Surgery, Samsung Medical Center, Seoul, South Korea; 5 Division of Nephrology, Department of Internal Medicine, College of Medicine, The St. Mary’s Hospital of Catholic University of Korea, Seoul, South Korea; 6 Division of Nephrology, Department of Internal Medicine, Inje University College of Medicine, Busan, South Korea; 7 Transplantation Center, Seoul National University Hospital, Seoul, South Korea; 8 East-West Bone & Joint Disease Research Institute, Kyung Hee University Hospital at Gangdong, Seoul, South Korea; 9 Department of Clinical Pharmacology and Therapeutics, College of Medicine, Kyung Hee University, Seoul, South Korea

## Abstract

Endocan is a water-soluble proteoglycan exclusively secreted by vascular endothelium. Endocan levels may be elevated in kidney transplant recipients experiencing antibody-mediated rejection (ABMR), which is characterized by vascular inflammation in transplanted kidney. We evaluated the clinical relevance of endocan as markers of microvascular inflammation in patients who underwent kidney transplantation. Plasma and urinary endocan levels were measured in 203 kidney transplant recipients and were compared across different etiologies of allograft dysfunction and various pathologic scores. Both plasma and urinary endocan levels were significantly higher in patients with acute ABMR than those in patients with normal pathology, acute tubular necrosis (ATN), acute pyelonephritis, BK virus associated nephropathy (BKVN), and T-cell mediated rejection (TCMR). Patients with chronic active ABMR also exhibited significantly higher plasma and urinary endocan levels than patients with long-term graft survival. Scores of glomerulitis and peritubular capillaritis, which are typical features of microvascular inflammation, were significantly elevated in patients with higher plasma and/or urinary endocan levels. Furthermore, plasma and urinary endocan levels could effectively discriminate ABMR from ATN, BKVN, and TCMR. Finally, patients exhibiting high urinary and plasma endocan levels in acute ABMR group showed significantly worse renal survival. Altogether, plasma and urinary endocan levels may serve as potential markers of microvascular inflammation in kidney transplant recipients.

## Introduction

Kidney transplantation (KT) is currently the treatment of choice for patients with end-stage renal disease. The one-year graft survival rate has gradually increased over the last two decades, reaching 96.5%^1^. However, allograft rejection remains a main cause of both early and late allograft dysfunction after KT despite substantial advances in immunosuppressive therapy. Timely diagnosis and prompt management of allograft rejection is often difficult in clinical practice since routine monitoring of serum creatinine levels is not sensitive with respect to detection of allograft rejection.

The vascular endothelium in the transplanted kidney is the major site of allograft rejection, especially in patients with antibody-mediated immune injury. Microvascular inflammation (MVI), characterized by histologic evidence of glomerulitis and peritubular capillaritis, is the basis for diagnosis of antibody-mediated rejection (ABMR). Several studies have demonstrated that these conditions are generally associated with poor allograft prognoses independent of other factors determining renal survival^2–11^. Currently, invasive renal biopsy is mandatory to demonstrate MVI, which carries substantial risks of complications. Numerous potential biomarkers of MVI are under investigation^12–18^; however, none can currently be used in clinical practice.

Endocan, or endothelial cell-specific molecule-1, is a water-soluble proteoglycan comprising amino acid polymers (molecular weight of 22 kDa) and a single dermatan sulfate chain^19^. The vascular endothelium is known to be the only site responsible for synthesis of endocan and its secretion into the blood. Previous studies have demonstrated that plasma endocan levels have potential as an endothelial activation marker^20–24^. Furthermore, a study demonstrated that endocan mRNA and protein expression levels were significantly elevated in patients with acute rejection after KT compared to those in healthy controls^25^. However, whether endocan can serve as a marker of MVI in kidney transplant recipients remains unknown. Given the role of the vascular endothelium in the process of ABMR, endocan levels may differ depending on the degree of vascular inflammation in renal allografts. The aim of our study was to evaluate the clinical relevance of plasma and urinary endocan levels as markers of MVI in kidney transplant recipients.

## Results

### Baseline demographic and clinical characteristics of the enrolled patients

A total of 203 kidney transplant recipients were recruited in our study, and their baseline clinical characteristics and laboratory data are shown in Table 1. The patients were classified into the following 8 different diagnostic groups: normal pathology (NP, n = 29), acute tubular necrosis (ATN, n = 17), acute pyelonephritis (APN, n = 7), BK virus associated nephropathy (BKVN, n = 22), acute T-cell mediated rejection (TCMR, n = 46), acute ABMR (n = 39), long-term graft survival (LTGS, n = 26), chronic active ABMR (n = 17). A detailed definition of each diagnostic group is provided in the Materials and methods section. These groups were further divided into two sets according to patient transplant vintages and were analyzed separately for each set to eliminate a confounding effect of transplant vintage; the short transplant vintage set included patients with NP, ATN, APN, BKVN, TCMR, and acute ABMR, and the long transplant vintage set included those with LTGS and chronic active AMBR.

**Table 1:** Baseline clinical characteristics and laboratory parameters of kidney transplant recipients according to diagnostic groups.

	Short transplant vintage set (n = 160)							Long transplant vintage set (n = 43)		
	NP (n = 29)	ATN (n = 17)	APN (n = 7)	BKVN (n = 22)	TCMR (n = 46)	Acute ABMR (n = 39)	*p*	LTGS (n = 26)	Chronic active ABMR (n = 17)	*p*
Age (years)	46.4 ± 10.9	40.5 ± 14.9	46.9 ± 12.5	47.2 ± 15.7	48.5 ± 13.4	48.4 ± 9.0	0.375	58.4 ± 9.2	48.4 ± 9.1	0.001
Sex (male, %)	21 (72.4)	10 (58.8)	4 (57.1)	17 (77.3)	31 (67.4)	21 (53.8)	0.431	14 (53.8)	9 (52.9)	0.954
Transplant vintage* (weeks)	35 ± 37	40 ± 111	45 ± 101	41 ± 40	97 ± 108	71 ± 93	0.029	913 ± 341	478 ± 249	< 0.001
Diabetes mellitus (n, %)	7 (24.1)	3 (17.6)	2 (28.6)	4 (18.2)	12 (26.1)	7 (17.9)	0.918	6 (23.1)	4 (23.5)	0.973
Hypertension (n, %)	17 (58.6)	27 (58.7)	3 (42.9)	11 (50.0)	27 (58.7)	24 (61.5)	0.840	12 (46.2)	8 (47.1)	0.954
ABO incompatible KT (n, %)	4 (13.8)	1 (5.9)	0 (0)	4 (18.2)	9 (19.6)	7 (17.9)	0.585	0 (0)	1 (5.9)	0.395
HLA mismatching (n)	4.0 ± 1.2	3.8 ± 1.6	3.9 ± 2.1	3.4 ± 1.8	3.7 ± 1.5	3.5 ± 1.8	0.751	3.4 ± 1.1	3.2 ± 1.5	0.721
**Indication for biopsy**										
Protocol biopsy (n, %)	23 (79.3)	9 (52.9)	2 (28.6)	0 (0)	0 (0)	0 (0)	0.330	N/A	0 (0)	N/A
Biopsy for causes (n, %)	6 (20.7)	8 (47.1)	5 (71.4)	22 (100)	46 (100)	39 (100)			17 (100)	
**Maintenance immunosuppression**										
Steroid (n, %)	29 (100)	15 (88.2)	6 (85.7)	21 (95.5)	43 (93.5)	38 (97.4)	0.104	13 (50.0)	15 (88.2)	0.020
Tacrolimus (n, %)	27 (93.1)	17 (100)	6 (85.7)	21 (95.5)	31 (67.4)	28 (71.8)	0.030	12 (46.2)	10 (58.8)	0.416
Cyclosporine (n, %)	2 (6.9)	0 (0)	1 (14.3)	0 (0)	13 (38.3)	8 (20.5)	0.031	12 (46.2)	5 (29.4)	0.272
Mycophenolate mofetil (n, %)	24 (82.8)	16 (94.1)	5 (71.4)	14 (63.6)	33 (71.7)	34 (87.2)	0.957	11 (42.3)	12 (70.6)	0.069
mTOR inhibitor (n, %)	2 (6.9)	0 (0)	0 (0)	3 (13.6)	2 (4.3)	3 (7.7)	0.835	1 (3.8)	5 (29.4)	0.028
eGFR (ml/min/1.73 m^2^)	72.0 ± 35.1	57.8 ± 22.0	35.0 ± 15.9	36.0 ± 13.3	33.4 ± 14.9	26.5 ± 12.0	<0.001	73.4 ± 17.3	29.9 ± 18.2	<0.001
Urine PCR (mg/gCr)	102 ± 163	81 ± 97	392 ± 351	264 ± 166	835 ± 1308	1157 ± 1208	0.002	145 ± 148	2456 ± 2192	<0.001
**Donor information**										
Donor Age (years)	46.5 ± 10.5	50.2 ± 9.4	54.3 ± 9.4	53.2 ± 8.7	50.0 ± 9.1	50.1 ± 11.1	0.245	34.3 ± 11.5	47.5 ± 14.5	0.002
Donor Sex (male, %)	17 (58.6)	8 (47.1)	5 (71.4)	11 (50.0)	23 (50.0)	16 (41.0)	0.629	11 (42.3)	12 (70.6)	0.069

*Transplant vintage is defined as the elapsed time between kidney transplantation and the time of biopsy or visit.

Abbreviations: NP: normal pathology; ATN, acute tubular necrosis; APN, acute pyelonephritis; BKVN, BK virus associated nephropathy; TCMR, T-cell-mediated rejection; ABMR, antibody-mediated rejection; LTGS, long-term graft survival; HLA, human leukocyte antigen; mTOR, mammalian target of rapamycin; eGFR, estimated glomerular filtration rate; PCR, protein-to-creatinine ratio; N/A, not applicable.

No differences in age or sex were observed in the short transplant vintage set, but transplant vintages were longer in patients with TCMR and acute ABMR than in those with NP, ATN, APN, and BKVN. Thirty-four (21.3%) samples were collected at the time of protocol biopsy, including 23, 9, and 2 samples from patients with NP, ATN, and APN, respectively. In contrast, all samples from patients with BKVN, TCMR, acute ABMR and chronic active ABMR were collected when graft biopsy was performed to evaluate graft dysfunction. The proportion of patients who underwent ABO-incompatible KT and the number of human leukocyte antigen (HLA) mismatches were not different across the groups. Patients with APN, BKVN, TCMR, and acute ABMR exhibited a significantly lower estimated glomerular filtration rate (eGFR) (72.0 ± 35.1 vs. 57.8 ± 22.0 vs. 35.0 ± 15.9 vs. 36.0 ± 13.3 vs. 33.4 ± 14.9 vs. 26.5 ± 12.0 ml/min/1.73 m^2^, NP vs. ATN vs. APN vs. BKVN vs. TCMR vs. acute ABMR; *p* < 0.001) and a higher urine protein-to-creatinine ratio (PCR) (102 ± 163 vs. 81 ± 97 vs. 392 ± 351 vs. 264 ± 166 vs. 835 ± 1308 vs. 1157 ± 1208 mg/gCr, NP vs. ATN vs. APN vs. BKVN vs. TCMR vs. acute ABMR; *p* = 0.002) than those with NP and ATN. In the long transplant vintage set, patients with LTGS were older and exhibited longer transplant vintages than those with chronic active ABMR. Finally, LTGS patients exhibited a higher eGFR and less proteinuria than patients with chronic active ABMR (73 ± 17 vs. 30 ± 18 ml/min/1.73 m^2^; *p* < 0.001 and 145 ± 148 vs. 2456 ± 2192 mg/gCr; *p* < 0.001; LTGS vs. chronic active ABMR, respectively).

### Comparisons of plasma and urinary endocan levels according to diagnosis

**Figure 1:**
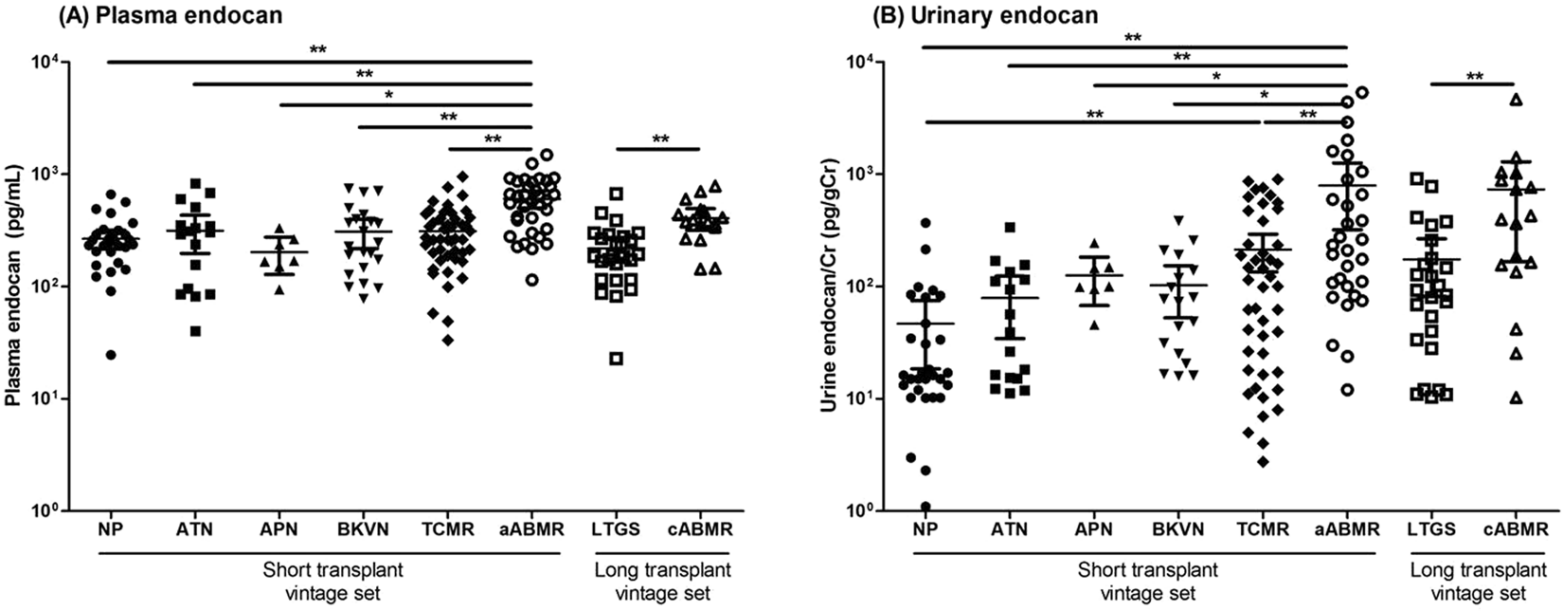
Endocan levels according to renal allograft status. (**A**) Plasma endocan levels were significantly higher in patients with acute ABMR than those in patients with NP, ATN, APN, BKVN, and TCMR. Patients with chronic active ABMR exhibited significantly higher plasma endocan levels than patients with LTGS. (**B**) Similar to plasma endocan levels, urinary endocan levels were significantly increased in patients with acute ABMR compared to those in the other groups in the short transplant vintage set. Patients with TCMR exhibited higher urinary endocan levels than patients with NP, and the levels were also higher in patients with chronic active ABMR than in patients with LTGS. *p < 0.05, **p < 0.005. Abbreviations: NP, normal pathology; ATN, acute tubular necrosis; APN, acute pyelonephritis; BKVN, BK virus associated nephropathy; TCMR, T-cell mediated rejection; aABMR, acute antibody-mediated rejection; LTGS, long-term graft survival; cABMR, chronic active antibody-mediated rejection.

Figure 1 presents patient plasma and urinary endocan levels according to renal allograft status. Plasma endocan levels were significantly elevated in patients with acute ABMR compared with those in patients with NP, ATN, APN, BKVN, and TCMR in the short transplant vintage set (271.5 ± 134.6 vs. 314.7 ± 228.9 vs. 207.2 ± 86.0 vs. 309.0 ± 204.6 vs. 311.2 ± 184.8 vs. 605.4 ± 306.5 pg/ml, NP vs. ATN vs. APN vs. BKVN vs. TCMR vs. acute ABMR; *p* < 0.001; Fig. 1A). In contrast, no differences in plasma endocan levels were found among the patients in the NP, ATN, APN, BKVN, and TCMR groups. Patients with chronic active ABMR exhibited significantly higher plasma endocan levels than patients with LTGS in the long transplant vintage set (219.0 ± 133.8 vs. 406.5 ± 171.7 pg/ml, LTGS vs. chronic active ABMR; *p* < 0.001; Fig. 1A).

Urinary endocan levels were also significantly increased in patients with acute ABMR compared to those in the other groups in the short transplant vintage set (45.1 ± 76.3 vs. 79.2 ± 87.3 vs. 113.1 ± 86.0 vs. 102.9 ± 101.1 vs. 258.6 ± 374.5 vs. 791.8 ± 1284.0 pg/mgCr, NP vs. ATN vs. APN vs. BKVN vs. TCMR vs. acute ABMR; *p* < 0.001; Fig. 1B). Furthermore, patients with TCMR exhibited higher urinary endocan levels than those with NP (*p* = 0.003). Finally, urinary endocan levels were significantly higher in patients with chronic active ABMR than those in patients with LTGS (174.5 ± 228.8 vs. 731.2 ± 1097.1 pg/mgCr, LTGS vs. chronic active ABMR; *p* = 0.006; Fig. 1B).

We also investigated the relationships between the abovementioned clinical parameters and endocan levels (Table 2). Age, transplant vintage, and the number of HLA mismatches were not associated with either plasma or urinary endocan levels. eGFR was negatively correlated with both plasma and urinary endocan levels; the correlation coefficient was much higher for urinary endocan levels (*r* = −0.195, *p* = 0.007 and = −0.425, *p* < 0.001, plasma endocan and urinary endocan, respectively). The urine PCR showed a positive correlation with urinary endocan levels but not with plasma endocan levels (*r* = 0.578, *p* < 0.001 and *r* = 0.183, *p* = 0.126, respectively). The presence of donor-specific antibody was not associated with either plasma or urinary endocan levels (data not shown).

**Table 2:** Correlation among plasma and urinary endocan levels and various clinical parameters.

	Plasma endocan (log_10_)		Urinary endocan (log_10_)	
	*r*	*p*	*r*	*p*
Age (years)	−0.051	0.489	0.061	0.416
Transplant vintage (week)	−0.081	0.261	0.104	0.155
HLA mismatching (n)	−0.031	0.665	−0.109	0.137
eGFR (ml/min/1.73 m^2^)	−0.195	0.007	−0.425	<0.001
Urine PCR (log_10_, mg/gCr)	0.183	0.126	0.578	<0.001

Abbreviations: HLA, human leukocyte antigen; eGFR, estimated glomerular filtration rate; PCR, protein-to-creatinine ratio.

### Associations between Banff scores and plasma and urinary endocan quartiles

Banff pathologic scores, adopted from the Banff 2007 classification^26^, of each diagnostic group are summarized in Table 3. Patients with TCMR exhibited prominent tubulitis (median score of 2) and interstitial inflammation (median score of 2), whereas patients with acute ABMR exhibited prominent glomerulitis (median score of 2) and peritubular capillaritis (median score of 2). Microvascular injuries were more prominent in patients with chronic active ABMR (median g score of 3 and median ptc score of 3) than in patients in other groups. Concomitant chronic tubulointerstitial changes were observed in patients with chronic active ABMR (median ct and ci scores of 2 and 2, respectively).

**Table 3:** Banff pathologic scoring categories according to renal allograft status.

	NP (n = 29)	ATN (n = 17)	APN (n = 7)	BKVN (n = 22)	TCMR (n = 46)	Acute ABMR (n = 39)	Chronic active ABMR (n = 17)
t	0 (0, 0)	0 (0, 0)	0 (0, 1)	1 (1, 2)	2 (1, 2)	0 (0, 2)	0 (0, 1)
v	0 (0, 0)	0 (0, 0)	0 (0, 0)	0 (0, 0)	0 (0, 0)	0 (0, 0)	0 (0, 0)
ti	0 (0, 0)	0 (0, 0)	1 (0, 2)	1 (0, 2)	2 (2, 3)	0 (0, 2)	2 (1, 2)
g	0 (0, 0)	0 (0, 0)	0 (0, 0)	0 (0, 0)	0 (0, 1)	2 (1, 3)	3 (2, 3)
ct	0 (0, 0)	0 (0, 0)	0 (0, 1)	0 (0, 1)	1 (0, 1)	1 (0, 2)	2 (1, 2)
cv	0 (0, 0)	0 (0, 0)	0 (0, 1)	0 (0, 1)	0 (0, 0)	0 (0, 1)	2 (1, 2)
ci	0 (0, 0)	0 (0, 0)	0 (0, 1)	0 (0, 1)	1 (0, 1)	1 (0, 1)	2 (1, 2)
cg	0 (0, 0)	0 (0, 0)	0 (0, 0)	0 (0, 0)	0 (0, 0)	0 (0, 0)	3 (2, 3)
ptc	0 (0, 0)	0 (0, 0)	0 (0, 0)	0 (0, 0)	0 (0, 0)	2 (2, 3)	3 (2, 3)
MVI	0 (0, 0)	0 (0, 0)	0 (0, 0)	0 (0, 0)	0 (0, 1)	4 (3, 5)	5 (4, 6)

Data expressed as medians (interquartile range).

Abbreviations: NP, normal pathology; ATN, acute tubular necrosis; APN, acute pyelonephritis; BKVN, BK virus associated nephropathy; TCMR, T-cell-mediated rejection; ABMR, antibody mediated rejection; t, tubulitis; v, intimal arteritis; ti, total interstitial inflammation; g, glomerulitis; ct, tubular atrophy; cv, chronic fibrous intimal thickening; ci, interstitial fibrosis; cg, transplant glomerulopathy; ptc, peritubular capillaritis; MVI, microvascular inflammation (sum of g score and ptc score).

**Figure 2:**
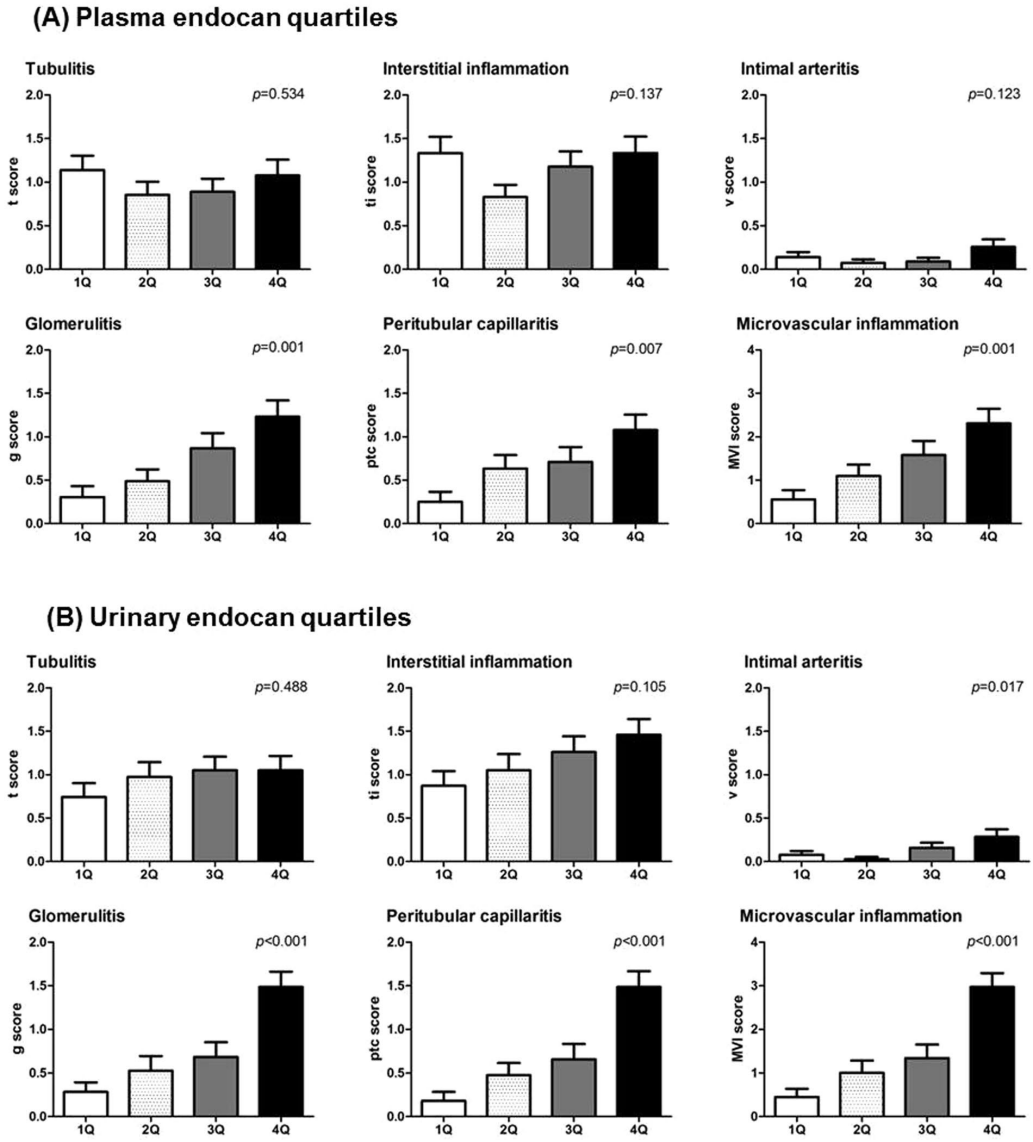
Association between plasma, urinary endocan levels and various Banff pathologic scores. Patients were divided into quartiles according to levels of (**A**) plasma and (**B**) urinary endocan, and various Banff pathologic scores were compared according to quartiles. Glomerulitis, peritubular capillaritis, and microvascular inflammation scores were elevated in the patients both in high plasma and/or urinary endocan quartiles, while tubulitis and interstitial inflammation scores were not.

The enrolled patients were subsequently organized into quartiles according to their plasma and urinary endocan levels, and various Banff pathologic scores were compared according to endocan quartiles (1Q, <189.0; 2Q, ≥189.0 and <280.3; 3Q, ≥280.3 and <450.8; 4Q, ≥450.8 pg/ml for plasma endocan levels and 1Q, <18.2; 2Q, ≥18.2 and <94.7; 3Q, ≥94.7 and <243.6; 4Q, ≥243.6 pg/mgCr for urinary endocan levels; Fig. 2). Neither tubulitis nor total interstitial inflammation scores were associated with plasma or urinary endocan quartiles. In contrast, glomerulitis, peritubular capillaritis, and MVI scores, calculated as the sum of glomerulitis and peritubular capillaritis score, were significantly elevated among patients in higher plasma and urinary endocan quartiles.

### Immunohistochemical and immunofluorescence staining of endocan in renal allograft tissues

**Figure 3:**
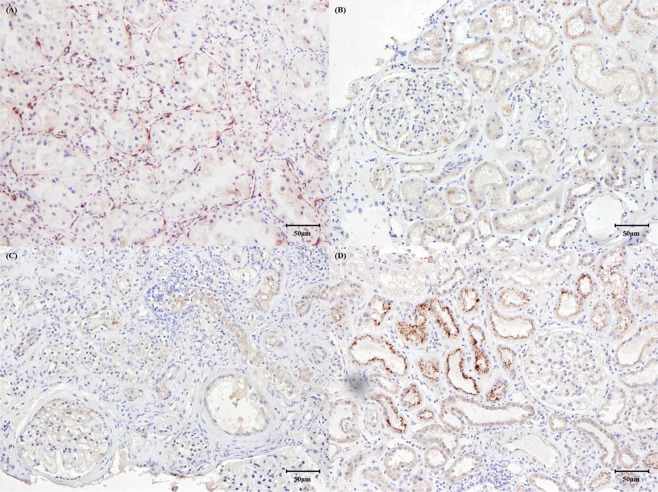
Immunohistochemistry with endocan antibody. (**A**) Tissues obtained from patients with clear cell renal cell carcinoma were selected as positive controls and stained with endocan. Diffuse immunoreactivity with endocan was found in vascular structures within tumors. (**B**,**C**) No immunoreactivity was observed in renal tissues obtained from patients with normal pathology and T-cell mediated rejection. (**D**) Positive immunostaining with endocan was observed in proximal tubular cells of tissues obtained from patients with acute antibody-mediated rejection. Other regions, including glomerulus, peritubular capillaries, and interstitium, were negative for endocan staining.

Figure 3 presents the results of the immunohistochemistry studies for endocan expression in renal tissues. As a positive control, tissues of patients with clear cell renal carcinoma were stained with endocan antibody^27^, and immunoreactivity was observed in the vascular endothelium of tumors (Fig. 3A). Endocan expression was not detected in tissues of patients with NP and TCMR (Fig. 3B and C). In contrast, endocan was expressed exclusively in proximal tubular epithelial cells in tissues of patients with acute AMBR (Fig. 3D). We did not observe any endocan expression in glomeruli or in large or small vessels, including peritubular capillaries, in these patients. Staining with isotype control confirmed that the endocan expression was not false positive (Supplementary Fig. 1)

As our immunohistochemical staining results were not concordant with the results of immunifluorescence staining of a previous study^25^, we further performed immunofluorescence staining of endocan to confirm our results (Supplementary Fig. 2). The pattern of endocan expression with immunofluorescence techniques was similar to the findings revealed by our immunohistochemistry studies; tissues of patients with NP and TCMR were negative for endocan immunofluorescence staining, and fluorescence activity was detected only in proximal renal tubular cells of tissues from patients with acute ABMR. Glomeruli and vessels were again not stained with endocan antibody.

### Receiver operating characteristic (ROC) curve analysis to evaluate the ability of plasma and urinary endocan levels to distinguish among ATN, BKVN, TCMR, and acute ABMR

**Figure 4:**
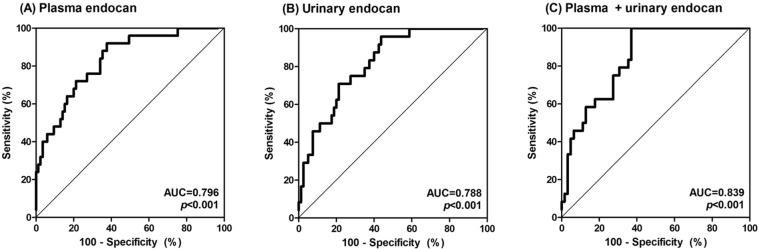
Receiver operating characteristics curves to evaluate the discriminative power of plasma and urinary endocan levels in distinguishing acute antibody-mediated rejection from acute tubular necrosis, BK virus associated nephropathy and T-cell mediated rejection. (**A**) Plasma endocan, (**B**) urinary endocan, and (**C**) the combination of plasma and urinary endocan levels.

We evaluated the diagnostic power of plasma and urinary endocan levels to distinguish acute ABMR from ATN, BKVN, and TCMR using the AUC, which was determined via ROC curve analysis (Fig. 4). This should be of particular interest for clinicians because these four conditions have indistinguishable clinical manifestations and the definitive diagnosis cannot be determined without graft biopsy. To eliminate the confounding effects of other variables, the ROC curves were generated by adjusting with age, sex, transplant vintage, the presence of diabetes mellitus and hypertension, ABO incompatibility, the number of HLA mismatching, and eGFR. The AUCs of plasma and urinary endocan levels were 0.796 and 0.788, respectively. The AUC value was increased to 0.839 after the integration of plasma and urinary endocan levels.

### Treatment modality and renal outcomes of patients

Patients received various combinations of immunosuppressive therapy after the diagnosis of rejection was confirmed (Supplementary Table 1). High-dose intravenous steroid therapy was given to the majority of patients diagnosed with rejection (78.3%, 84.6%, and 76.5% of patients with TCMR, acute ABMR, and chronic active ABMR, respectively). A substantial portion of patients with ABMR additionally received intravenous immunoglobulin (79.5% and 58.8% of patients with acute ABMR and chronic active ABMR, respectively) and rituximab (74.4% and 70.6% of patients with acute ABMR and chronic active ABMR, respectively) to eliminate alloreactive antibodies. Therapeutic plasma exchange was also frequently performed in the acute ABMR group (79.5%). The diagnoses of NP and ATN did not alter the prescription patterns of immunosuppressants.

**Figure 5:**
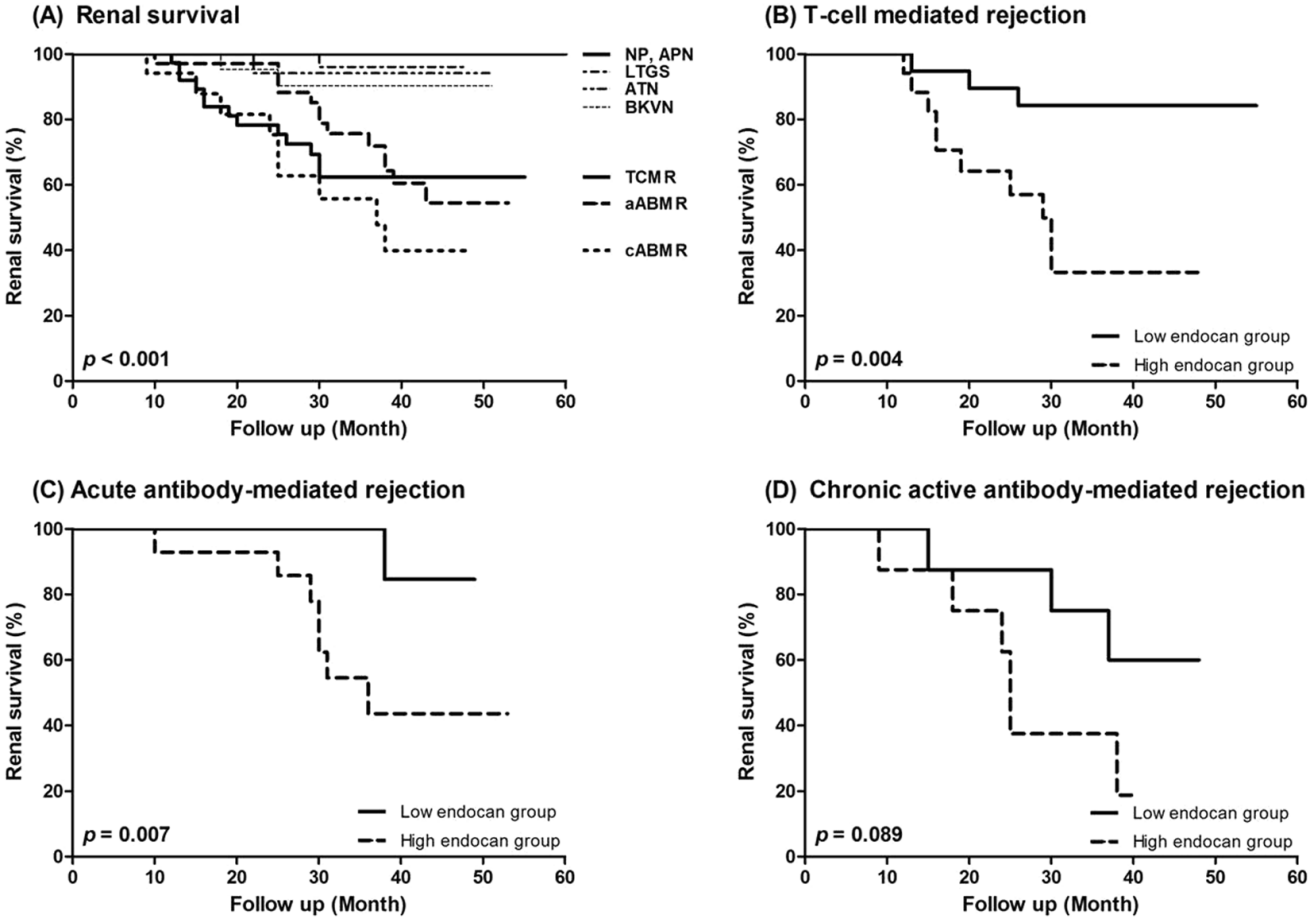
Renal allograft survival of enrolled patients. (**A**) Renal survival according to the diagnosis. (**B–D**) Renal survival according to plasma and urinary endocan levels in (**B**) acute T-cell mediated rejection, (**C**) acute antibody-mediated rejection, and (**D**) chronic active antibody-mediated rejection group. Plasma and urinary endocan levels were integrated by binary regression analysis.

Finally, renal outcomes were analyzed according to the diagnosis and endocan levels (Fig. 5). Again, the survival curves were created after multivariate adjustments described above to avoid possible confounding effects. Patients diagnosed with any type of rejection experienced significantly worse renal allograft survival than those in the other groups (*p* < 0.001; Fig. 5A). Interestingly, the combination of plasma and urinary endocan levels was associated with allograft survival in both TCMR and ABMR groups; patients with higher endocan levels in TCMR and acute ABMR group exhibited significantly poorer renal survival than those with lower endocan levels (*p* = 0.004 and *p* = 0.007, respectively; Fig. 5B and C). A similar finding was observed in patients with chronic active ABMR, although the difference was a weak trend toward significance (*p* = 0.089; Fig. 5D).

## Discussion

In this study, we demonstrated that plasma and urinary endocan levels were higher in patients with ABMR than those in patients with NP, ATN and TCMR. Furthermore, MVI scores were positively correlated with both plasma and urinary endocan levels. In contrast, neither tubulitis severity nor interstitial inflammation severity was associated with plasma or urinary endocan levels, indicating that endocan is a specific marker of endothelial injury. These findings are consistent with our hypothesis, as the vascular endothelium is the major site of immunologic reactions in ABMR, but not in ATN or TCMR. Li *et al*. observed elevated plasma endocan levels in patients experiencing acute rejection after KT^25^. However, their study did not include pathologic analyses and lacked data regarding urinary endocan levels. We measured both urinary and plasma endocan levels, which provided new insight into the physiology and renal excretion of endocan. Furthermore, we were able to elucidate the relationship between endocan levels and MVI by analyzing pathologic features of renal allografts in patients with elevated endocan levels.

The subject of MVI in KT has received increasing attention over the past 10 years. The two main histologic features of ABMR have traditionally been MVI and diffuse C4d staining. However, evidence has shown that C4d-negative and MVI-positive kidney transplant recipients exhibited poor allograft survival rates, which were similar to of the findings in patients with C4d-positive ABMR^2,3,5,28^, leading to revisions of the diagnostic criteria for ABMR. The new criteria acknowledge the existence of C4d-negative ABMR and emphasize the presence of MVI^10^. Accordingly, biomarkers of MVI may provide clinicians with valuable information for predicting statuses of renal allografts and diagnosing rejection. Numerous studies have investigated variables that may serve as useful biomarkers of endothelial injury, including syndecan-1, von Willebrand factor, and angiopoietin-1 and 2, as well as selectins, cell adhesion molecules, circulating endothelial cells, and microRNAs^29–32^. However, these variables have not been sufficiently investigated or validated as markers of MVI in kidney transplant recipients.

To identify a potential marker of MVI, we focused on endothelial injuries in renal allografts. Allograft endothelial cells express various antigens on their surfaces that are recognized by the host immune system immediately after transplant surgery. Without appropriate immunosuppression, preformed and/or induced allo-antibodies can bind to the allograft endothelium, provoking endothelial inflammation^33^. One study reported that leukocytes sensitized by alloantigens induced intercellular adhesion molecule (ICAM)-1, vascular cell adhesion molecule (VCAM)-1 and endothelium leukocyte adhesion molecule-1 overexpression on allograft endothelial cells, all of which are involved in leukocyte adhesion to and migration into transplanted kidneys. These immunologic reactions may contribute to sustained inflammation within and leukocyte recruitment to the allograft endothelium, leading to vascular inflammation and transplant rejection^34^. Interestingly, in kidney transplant recipients exhibiting LTGS, renal endothelial cells were resistant to complement-induced cell lysis^35^. Taken together, these data indicate that maintaining endothelium homeostasis is crucial for maintaining graft function. Therefore, we speculated that endocan may be useful as a noninvasive marker of vascular endothelial inflammation.

Previous studies have demonstrated conflicting results regarding the biological functions of endocan. Lee *et al*. demonstrated that endocan upregulated cell adhesion molecules, such as ICAM-1, VCAM-1 and E-selectin^20^. These authors also demonstrated that endocan activated the nuclear factor-kappa B pathway, a key mediator of inflammatory reactions. Taken together, their findings suggested that plasma endocan could elicit vascular inflammation via endothelial activation and cell recruitment. In contrast, Bechard *et al*. demonstrated that endocan could bind directly to lymphocyte function-associated antigen-1 (LFA-1), which is located on the surface of human lymphocytes^36^. This finding suggested that endocan could block lymphocytes recruitment by interfering with the interaction between LFA-1 and ICAM-1, thereby attenuating vascular inflammation. Whether endocan exerts pro-inflammatory or anti-inflammatory effects on the vascular endothelium remains to be determined. Regardless of its biological functions, plasma endocan has been shown to be correlated with inflammation severity and poor survival in various diseases, such as coronary artery disease, malignancy, chronic kidney disease, IgA nephropathy and sepsis^37–45^.

Importantly, our results could provide insight regarding the renal metabolism of endocan. The origin of endocan in urine is uncertain, as vascular endothelial cells are the only cells responsible for secreting endocan, as described above. Moreover, the negatively charged glomerular basement membrane can prevent endocan from escaping from blood into urine, as dermatan sulfate in endocan is a negatively charged glycosaminoglycan^19^. Two hypotheses explaining how endocan is present in urine have been proposed. First, endocan may leak into the urine through the disrupted glomerular basement membrane after glomerular injury, such as rejection. Second, endocan may be secreted directly into the urine by injured renal tubular cells. Our data indicated that patients with high urinary endocan levels exhibited more severe glomerulitis scores than patients with low urinary endocan levels; however, tubulitis scores were not correlated with urinary endocan levels. We also noted a positive correlation between urinary endocan levels and urine PCR, a marker of glomerular injury. Taken together, our results suggest that urinary endocan originates in the plasma and enters the urine by leaking through damaged glomeruli rather than being secreted by damaged renal tubular cells.

Plasma endocan levels were elevated with increasing kidney function deterioration in our analysis, which is consistent with reports from previous studies^40,46^. Therefore, elevated plasma endocan levels may be a consequence of decreased renal endocan clearance rather than increased endocan excretion by damaged endothelial cells. However, urinary endocan levels were also elevated proportionately to the declines in renal function noted in our study, indicating that patients with severe renal dysfunction tend to exhibit greater urinary endocan loss. Moreover, we found that ABMR patients with higher plasma and urinary endocan levels were likely to have worse renal outcomes independent of baseline renal function (Fig. 5C and D), which was not demonstrated when either plasma or urinary endocan levels were used as single markers (data not shown). We speculated that plasma and urinary endocan levels could reflect MVI within the whole transplanted kidney; therefore, the integration of both endocan levels could provide additional information regarding the intragraft pathology.

We revealed that immunohistochemical staining of endocan was positive only in patients with acute ABMR. However, endocan expression was observed exclusively in proximal tubular cells, while staining was negative in the glomerulus and peritubular capillaries. In contrast to our findings, Li *et al*. reported positive immunofluorescence staining of endocan antibody in renal tissues of patients experiencing acute rejection^25^, and the expression was localized in glomeruli rather than renal tubular cells or peritubular capillaries. Given the roles of endothelial injuries in the pathogenesis of acute ABMR, it is highly likely that endocan is secreted from damaged endothelial cells in the transplanted kidney. Nonetheless, we concluded that endocan could not be detected in the vascular endothelium of the transplanted kidney by either immunohistochemical or immunofluorescence staining. The reasons for negative endocan expression in glomeruli and peritubular capillaries were unclear in this study. Positive immunohistochemical staining of endocan was primarily found in the analysis of the tissues obtained from patients with malignancy^37–39,42,47–49^. Furthermore, except for one study^25^, we could not find any report indicating positive microvascular endocan expression induced by endothelial inflammation. Based on our findings and review of the literature, we concluded that immunohistochemical evidence of endocan expression in the vascular endothelium could be observed exclusively in neoplastic vessels, but not in injured vessels. Considering the low molecular weight of endocan (less than 50 kDa) and the characteristic role of proximal tubular cells regarding proteins that have not been retained by the glomeruli, the unexpected positive staining in proximal tubular cells of patients with ABMR may be due to endocytosis of the delivered endocan to proximal tubules.

Several limitations of this study should be mentioned. This was a retrospective cohort study, and future investigations should be performed to validate the relevance of endocan as a diagnostic and prognostic marker. Additionally, some medications can influence endocan levels by changing endothelial integrity, such as angiotensin receptor blockers, calcium channel blockers and statins^50,51^. We need to investigate the effects of other drugs, such as immunosuppressive agents, on plasma and urinary endocan levels. Finally, data regarding endocan levels after treatment of rejection was not measured in this study.

In conclusion, our results demonstrated that plasma and urinary endocan levels have potential as markers of MVI in kidney transplant recipients. We expect that further prospective trials will confirm whether endocan levels can provide new insight into the overall status of renal allograft, especially the degree of MVI, and complement the findings of renal biopsy, which is a snapshot of a restricted area in the transplanted kidney.

## Materials and Methods

### Study population

We retrospectively enrolled kidney transplant recipients from Kyung Hee University Hospital at Gangdong, Kyung Hee University Medical Center, Seoul St. Mary’s Hospital, Kyungpook National University Hospital, Samsung Medical Center, Busan-Baik Hospital and Seoul National University Hospital between August 2012 and December 2016. Patients were recruited at the time of graft biopsy except LTGS group. Graft biopsy was performed either to evaluate the cause of graft dysfunction, including increased serum creatinine levels and/or proteinuria (biopsy for cause) or for surveillance of renal allograft status (protocol biopsy). Only two centers performed protocol biopsies for the patients who provided written consent (3 weeks and 6 months after KT in Kyung Hee University at Gandong, and 3 months after KT in St. Mary’s Hospital of Catholic University). Finally, patients with LTGS, defined as having stable graft function and no evidence of proteinuria for more than 10 years after KT (eGFR > 50 ml/min/1.73 m^2^ and urine PCR < 300 mg/gCr), were recruited in this study.

Information regarding age, sex, the presence of diabetes mellitus and hypertension, immunologic status, serum creatinine levels and urine PCR was obtained from each patient at the time of graft biopsy for the biopsy cohort, or during a visit to the outpatient clinic for the LTGS group. Renal function was assessed by the eGFR calculated using the Chronic Kidney Disease Epidemiology Collaboration (CKD-EPI) formula^52^. The institutional review board of each hospital approved this study (IRB No 2012-01-030), and informed consent was obtained from all patients. No organs/tissues were procured from prisoners, and all methods were performed in accordance with the relevant guidelines and regulations.

### Pathologic diagnoses and classifications according to renal allograft status

Patients who underwent graft biopsy were classified according to the Banff classification as described above. Patients with NP were defined by serum creatinine levels below 1.2 mg/dL and no histologic evidence of rejection, calcineurin inhibitor toxicity, acute tubular necrosis, tubulointerstitial inflammation, vascular injury or glomerulonephritis. ATN was diagnosed when graft biopsy revealed features of moderate or severe ATN in the absence of other graft injuries. APN was diagnosed by pyuria, the presence of >10^5^ CFU/mL on urine culture, and the evidence of neutrophil infiltration in tubulointerstitium. BKVN was diagnosed as previously described^53^. Finally, TCMR, acute ABMR, and chronic active ABMR were diagnosed based on the Banff 2007 criteria^26^. Patients exhibiting features of both TCMR and ABMR, subclinical rejection, calcineurin inhibitor toxicity, and glomerulonephritis were excluded from this study.

All scores and pathologic diagnoses were determined in accordance with the scoring categories^26^ and included tubulitis (t), total interstitial inflammation (ti), intimal arteritis (v), glomerulitis (g), tubular atrophy (ct), interstitial fibrosis (ci), chronic transplant glomerulopathy (cg) and peritubular capillaritis (ptc). The MVI score was defined as the sum of the g score and the ptc score^10^.

### Collection of study samples and measurement of endocan levels

Plasma and urinary samples were collected early in the morning on the day of the patients’ graft biopsy procedures. Samples were also randomly collected from the patients in the LTGS group during routine follow-up visits to the outpatient clinic. Peripheral blood samples were collected in EDTA-treated tubes, and the cells were removed from these samples via centrifugation for 30 minutes at 2000 g at room temperature. The supernatant was designated the plasma, which was separated into 1-ml aliquots and stored at −80 °C in a deep freezer until analysis. Urinary samples were collected in 50-ml sterile conical tubes and centrifuged for 20 minutes at 2000 g at room temperature. Then, the supernatants were placed into tubes and stored at −80 °C in a deep freezer until analysis. All samples were processed and stored within 1 hour of collection.

Plasma and urinary endocan concentrations were analyzed via enzyme-linked immunosorbent assay (ELISA) using commercial kits (Boster Biological Technology, Pleasanton, CA) in accordance with the manufacturer’s instructions. The absorbance was measured at 450 nm on a spectrophotometer. Urine creatinine concentrations were analyzed by ELISA using commercial kits in accordance with the manufacturer’s instructions, and urinary endocan levels were adjusted by urine creatinine concentrations and expressed as ratios of urinary endocan/urine creatinine (pg/mgCr).

### Immunohistochemical staining of endocan

Immunohistochemistry procedures were performed on 3-μm tissue sections using a Bond Polymer Intense Detection System (Vision BioSystems, Hingham, MA), in accordance with the manufacturer’s instructions, with minor modifications. Briefly, 3-μm sections of formalin-fixed, paraffin-embedded tissues were deparaffinized with Bond Dewax solution (Vision BioSystems), and antigen retrieval was performed using Bond ER solution (Vision BioSystems) for 30 min at 100 °C. Endogenous peroxidase was quenched by incubation with hydrogen peroxide for 5 min. The sections were then incubated for 15 min at ambient temperature with primary polyclonal antibodies to endocan (Abcam, catalog No. ab224591, Cambridge, MA) using a biotin-free polymeric horseradish peroxidase-linked antibody conjugate system in a Bond-maX automatic slide stainer (Vision BioSystems). Rabbit IgG isotype control (Abcam, catalog No. ab37415) staining was performed as a negative control. Cell nuclei were counterstained with hematoxylin.

### Immunofluorescence staining of endocan

Immunofluorescence staining was applied to 4-μm sections of formalin-fixed, paraffin-embedded tissues. Deparaffinization, antigen retrieval, and blocking of endogenous peroxidase activity were performed in the tissue samples using the same methods described above. Then, the samples were blocked with 1% bovine serum albumin and incubated with antibodies to endocan (Abcam, catalog No. ab103590) for 2 hours. After washing with PBS, the samples were re-incubated with secondary antibodies conjugated with Alexa Fluor 488 (Life Technologies, Gaithersburg, MD) for 1 hour. The cells were counterstained with DAPI to delineate the nuclei, and the sections were examined via confocal microscopy (LSM-700; Carl Zeiss, Jena, Turingia, Germany).

### Treatment and renal outcome of patients

Patients received appropriate immunosuppressive treatment according to the guidelines of each hospital after the diagnosis of rejection. Renal function was assessed routinely in the outpatient clinics, and the renal outcome was assessed by progression to end-stage renal disease, defined as a condition requiring either dialysis or re-transplantation.

### Statistical analysis

All statistical analyses were performed using SPSS for Windows, version 20.0 (IBM Corp, Armonk, NY). Demographic and clinical data are expressed as the mean ± standard deviation or as the numbers of patients and percentages. Analysis of variance (ANOVA) with Bonferroni post hoc analysis, independent t-tests and chi-square tests were used for comparisons of baseline characteristics and laboratory findings as appropriate. Between-group analyses of plasma and urinary endocan levels were performed after log-transformation. We used Pearson simple correlation analyses to evaluate relationships between endocan levels and other parameters. Banff pathologic scores are expressed as the mean ± standard error of the mean using bar graphs, and comparisons between these scores were assessed via ANOVA.

ROC curves were created, and AUC was calculated to compare the discriminative power of plasma and urinary endocan levels to distinguish acute ABMR from ATN and TCMR. For the assessment of renal outcomes, Kaplan-Meier survival curves were analyzed. At the time of generating ROC and survival curves, covariate adjustments for logistic regression analyses were performed to eliminate possible confounding effects of other variables. *p*-values less than 0.05 were considered statistically significant.

## Supplementary information

Supplementary data

## Data Availability

The datasets generated during and/or analyzed during the current study are available from the corresponding author on reasonable request.
