# Supplementary material for: Endocan as a marker of microvascular inflammation in kidney transplant recipients
Source: Sci Rep. 2019 Feb 12;9:1854. doi: 10.1038/s41598-018-37975-9 (PMC6372712; doi:10.1038/s41598-018-37975-9)

Endocan as a marker of microvascular inflammation in kidney transplant recipients

Yu Ho Lee[1†], Se Yun Kim[1†], Haena Moon[1], Jung-Woo Seo[1], Dong-Jin Kim[1], Seon Hwa Park[1], Yang Gyun Kim[1], Ju-Young Moon[1], Jin Sug Kim[1], Kyung-Hwan Jeong[1], Sung-Jig Lim[2], Chan-Duck Kim[3], Jae Berm Park[4], Byung Ha Chung[5], Yeong Hoon Kim[6], Jaeseok Yang[7], Hyung-In Yang[8], and Kyoung Soo Kim[8, 9*], Sang-Ho Lee[1*]

[1]Division of Nephrology, Department of Internal Medicine, [2]Department of Pathology, Kyung Hee University, Seoul; [3]Division of Nephrology, Department of Internal Medicine, Kyungpook National University Hospital, Daegu; [4]Department of Surgery, Samsung Medical Center, Seoul; [5]Division of Nephrology, Department of Internal Medicine, College of Medicine, The St. Mary's Hospital of Catholic University of Korea, Seoul; [6]Division of Nephrology, Department of Internal Medicine, Inje University College of Medicine, Busan; [7]Transplantation Center, Seoul National University Hospital, Seoul; [8]East-West Bone & Joint Disease Research Institute, Kyung Hee University Hospital at Gangdong, Seoul; [9]Department of Clinical Pharmacology and Therapeutics, College of Medicine, Kyung Hee University, Seoul

Supplementary table 1. Treatment modality of patients

	TCMR (n=46)	Acute ABMR (n=39)	Chronic active ABMR (n=17)
**Steroid pulse therapy (n, %)**	36 (78.3)	33 (84.6)	13 (76.5)
**Anti-thymocyte globulin (n, %)**	13 (28.3)	8 (20.5)	0 (0)
**Intravenous immunoglobulin (n, %)**	7 (15.2)	31 (79.5)	10 (58.8)
**Rituximab (n, %)**	5 (10.9)	29 (74.4)	12 (70.6)
**Therapeutic plasma exchange (n, %)**	3 (6.5)	31 (79.5)	5 (29.4)

Abbreviations: TCMR, T-cell-mediated rejection; ABMR, antibody-mediated rejection.

Supplementary figure 1. Immunohistochemical staining with isotype control. As a negative control, kidney tissues obtained from the same patients were stained with isotype antibody; the result confirmed that the endocan expression was not false positive. (A) Tissues of patients with clear cell renal carcinoma, (B) normal pathology, (C) acute T-cell mediated rejection, and (D) acute antibody-mediated rejection.

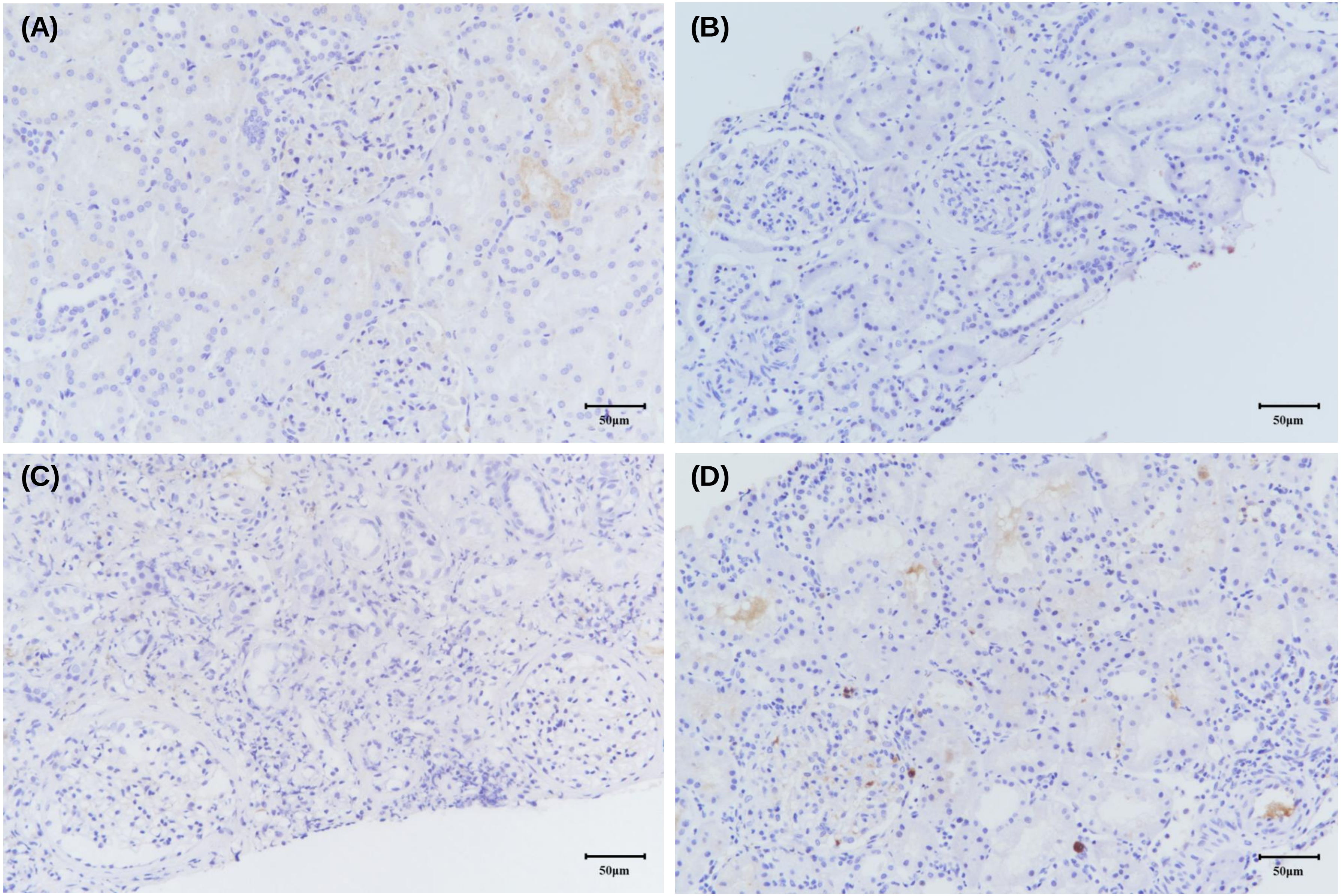

Supplementary figure 2. Immunofluorescence staining of endocan. Renal allograft tissues obtained from patients with normal pathology (NP), T-cell mediated rejection (TCMR), and acute antibody-mediated rejection (ABMR) were stained with endocan antibody. Similar to our immunohistochemical study, the tissues of patients with NP and TCMR were negative for endocan immunofluorescence staining. Endocan expression was positive only in proximal tubular cells of the tissues obtained from patients with acute AMBR.

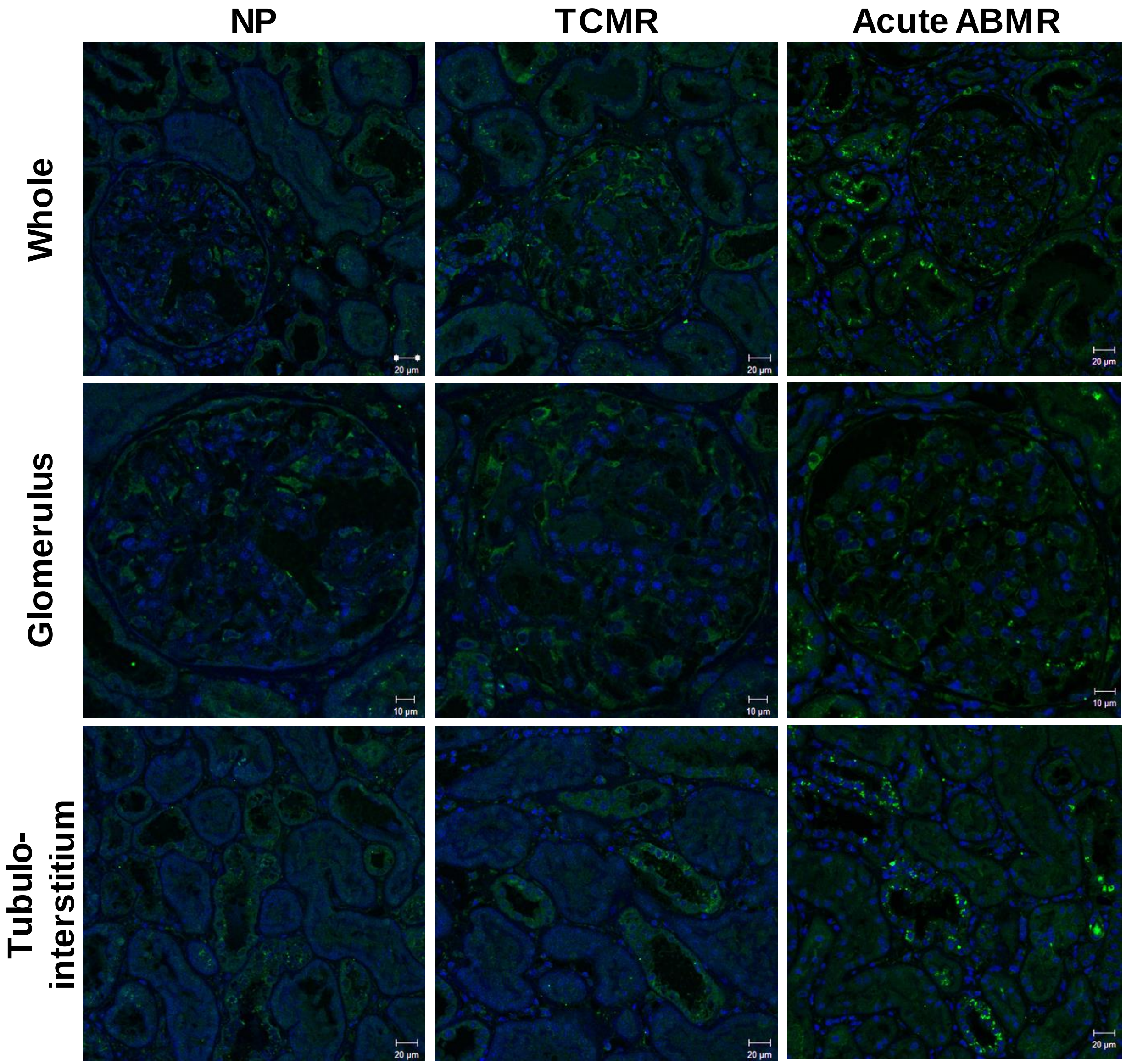

Supplement: Supplementary file 1 — Supplementary data [file 41598_2018_37975_MOESM1_ESM.pdf]
